# Commentary: The COVID‐19 pandemic and its impact on adolescent development: Embracing a more ecological perspective

**DOI:** 10.1111/jora.13036

**Published:** 2024-11-03

**Authors:** Nora Wiium

**Affiliations:** ^1^ Department of Psychosocial Science University of Bergen Bergen Norway

**Keywords:** adolescent development, civic engagement, COVID‐19 pandemic, ecological perspective, family and peer relationships, school experiences

## Abstract

To assess the impact of the COVID‐19 pandemic on young people's functioning, relationships, and well‐being, four systematic reviews were put together to shed light on school experiences, family and peer relationships, and civic engagement during the pandemic. The reviews presented research findings on the protective role of several personal and contextual resources including intrinsic motivation, self‐efficacy, family, school, peer, and community support, as well as the harming effect of risk factors, such as poor mental health, COVID‐19‐related stressors, and technological challenges, thus highlighting the significant role of both personal and contextual factors in adolescent development and well‐being. Equally important, the research findings collectively suggested an ecological perspective of the determining factors, although the focus was largely on factors in immediate contexts (family, school, peers, and local community). Adopting a more holistic approach that also considers factors in other ecological contexts (e.g., partnership between immediate contexts, the influence of cultural values and norms along with educational and developmental transitions) can be crucial in addressing the specific needs of young people across diverse contexts and cultures during a pandemic and in general. In addressing their needs, the ever‐growing digital space of young people can be utilized to connect them to services and supportive networks in their contexts including distal ones.

Theory and research suggest that human development is multidetermined. For adolescents and emerging adults, key factors include those related to family, school, and local community. Indeed, the ecological systems model explains how factors in the immediate contexts of young people (microsystem) along with personal factors (i.e., personal agency and other intrapsychic factors) can determine the course of their development (Bronfenbrenner & Morris, 2006). The factors in the microsystem do not influence development independently; their influence can be reinforced or weakened through interaction with other factors (mesosystem). Moreover, the ecological systems model proposes that factors in more distal contexts (exo‐ and macrosystem), for example, government policies, social media, and social services, as well as cultural values, social norms, and traditions can influence human development through the resources and opportunities they offer or constrain.

Inspired by the implications of developmental systems meta‐theories like Bronfenbrenner's ecological systems model and others (Overton, 2015), Positive Youth Development (PYD) theoretical frameworks posit that positive development is the outcome of an optimal alignment of the strengths and competences of young people with the resources and opportunities in their contexts (Benson et al., 2011; Lerner et al., 2015). The ensuing, so called adaptive developmental regulation (from a relational developmental systems meta‐theoretical standpoint; Overton, 2015) from this alignment is expected to promote youth thriving and enrichment of the communities in which youth live. Thus, young people are not only equipped to act, but they can also be given the opportunity to participate and lead initiatives that concern their development and that of their community. When the strengths‐resources alignment does not benefit youth or their community, there can be gaps and missed opportunities for growth of strengths and thriving at the individual and community levels.

At the beginning of 2020, when the World Health Organization declared a Public Health Emergency of International Concern (due to the spread of a novel coronavirus) and soon after upgraded the epidemic to pandemic, governmental restrictions, school closures, and other pandemic guidelines that followed not only affected the daily lives and routines of young people around the globe, but they also restricted the resources and opportunities available within youth contexts. The odds of missed opportunities for development and youth contribution along with negative cascading effects on various developmental processes and outcomes of young people were thus more plausible. To assess the impact of the COVID‐19 pandemic on young people's functioning, relationships, and well‐being, a Special Issue comprising four systematic reviews was put together by *The Journal of Research in Adolescence* (*JRA*). The review articles systematized and synthesized knowledge to shed light on the impact of the pandemic on school experiences, family and peer relationships, as well as youth involvement in civic engagement.

Adapting a global perspective in their inclusion of publications, the reviews offered insight into developmental assets but also risk factors found in the immediate contexts of diverse youth that have significant implications for their development and well‐being. Across the reviews (see review articles by Benner et al., 2024; Campione‐Barr et al., 2024; Magis‐Weinberg et al., 2024; Schoon et al., 2025), research on the protective role of several internal and external developmental assets (e.g., intrinsic motivation, self‐efficacy, family, school, peer, and community support), as well as the harming effect of risk factors (e.g., poor mental health, COVID‐19‐related stressors, and technological challenges) was presented. Collectively, the reviews presented findings that confirmed the ecological perspective of the determining factors of youth development in a health emergency, although the focus was largely on factors located in the micro‐ and exo‐system (family, school, peers, local community, and social media). Research investigations that also consider factors located in the mesosystem (e.g., partnership between family and school) and factors within the macrosystem (e.g., the influence of cultural values and social norms) can be essential to inform effectual and beneficial youth initiatives and interventions inside as well as outside of non‐normative times of crisis.

When addressing the conditions needed for youth thriving and contribution, the PYD perspective emphasizes the importance of both internal and external developmental assets (personal and contextual resources). Interestingly, key factors found to contribute to civic engagement in diverse youth during the pandemic included both individual and community‐level or institutional factors (see review article by Schoon et al., 2025), thus affirming PYD's take on what drives positive development and contribution among young people. Nonetheless, as Schoon and colleagues' review also revealed, this was not the case in all contexts, as in some developing and disadvantage contexts, youth had to rely solely on their personal strengths and skills; an inadequate situation, which in line with PYD may not favor youth thriving and contribution.

Extending the argument of the ecological perspective of human development, differences in the findings related to the functioning, relationships, and well‐being of adolescents and emerging adults may not only be due to differences across families, schools, and local communities (microsystem). The differences in findings may be a product of other distal factors in the ecological systems model, such as cultural values and social norms, as well as social services (exo‐ and macrosystem). While the studies reported in the reviews considered some of these distal factors, for example, social media, community and institutional services and support (Magis‐Weinberg et al., 2024; Schoon et al., 2025), taking a more inclusive ecological approach that also considers relevant factors at the meso‐, exo‐, and macrosystem levels, such as partnership between family, school, and local community, social services, and the influence of cultural values and social norms, can enhance our understanding of what influences development and well‐being among young people during a pandemic but also in general. In a systematic review of family and school relationships during the pandemic, Carrión‐Martínez et al. (2021) argued that students' needs brought about by the shift to online learning can be effectively met if partnership between family, students, and school is enhanced. Padilla‐Walker et al.'s (2022) study of cultural values and mental health in emerging adults during the pandemic indicated how cultural values, especially collectivism, were indirectly associated with lower levels of depression through prosocial behavior toward family members.

Furthermore, variations in findings across the reviews can be attributed to the characteristics of young people and/or the conditions of their contexts. Referring to the adaptive developmental regulation processes, PYD scholars described how youth thriving and well‐being are facilitated through a dynamic bidirectional interaction between an active, engaged, and competent person and a receptive, supportive, and nurturing ecology (Benson et al., 2006; Lerner et al., 2015). Thus, why some students thrived with online education while others did not (see review article by Benner et al., 2024) could be due to factors and their interaction at various levels (e.g., individual and/or contextual). For students who succeeded with online education, a nurturing teaching context and a home learning environment that was conducive to online learning were some of the factors that contributed to positive school experiences. Similarly, several personal factors like intrinsic motivation and self‐efficacy were found to be positively associated with school experiences and outcomes, while other personal and contextual factors like poor mental health, COVID‐19‐related stressors, technological challenges, and limited interaction and support from teachers and peers were negative predictors (Benner et al., 2024). While these findings appear to confirm the importance of an adaptive developmental regulation for positive development, it also suggests that in health emergencies, where drastic changes in the educational settings and structures of young people are inevitable, attention to supporting individual level mastery skills that can help them learn and solve problems as well as nurturing immediate contexts that can provide support, opportunities, and services, can ultimately enhance young people's school experiences and other developmental outcomes.

In addition to the four ecological systems (micro‐, meso‐, exo‐, and macrosystem) earlier referred to, the chronosystem, which focuses on the role of time in human development, is a fifth system in the ecological systems model. The different educational and developmental transitions that young people had to go through, in common with the challenges brought about by the pandemic, all constitute elements of time that can influence adolescent development. Edge et al.'s (2024) study on the transition from primary to secondary school indicated a negative impact of the COVID‐19 pandemic on pupils' social skills and emotional well‐being. In another study of two high school graduation cohorts: 2020 and 2021, Sandner et al. (2023) observed that during transitions to post‐secondary education, although school closures had a positive immediate effect on students' well‐being, well‐being strongly declined over the course of the pandemic, especially among the 2021 graduation cohort. Extending the research on youth ecology to include elements of time or factors in the chronosystem may help unravel the effects of these transitions and challenges so that the development and well‐being of young people can be promoted effectively.

Digital space is becoming a crucial context for youth development and could be considered even more so during the pandemic. Studies involved in the reviews that considered this context consistently reported an increase in social media use with COVID‐19‐related experiences, with peer interaction largely moving online (see review articles by Campione‐Barr et al., 2024; Magis‐Weinberg et al., 2024). Other studies across the reviews revealed how digital space became a relevant platform for civic engagement activities (see review article by Schoon et al., 2025). Indeed, so important is digital space to youth development that scholars have proposed that a modification be made to the ecological systems model to differentiate between physical and virtual microsystems (Navarro & Tudge, 2023). While this differentiation at the microsystem may be necessary, using digital space as a mass‐media strategy (exosystem) to communicate, collaborate, and connect youth with their distal contexts (e.g., youth initiatives and programmes at the regional, national, and international level) can be equally important. To the extent that digital space can transcend boundaries of contexts, cultures, and countries, it can become an important arena where young people around the globe can engage with each other but also with organizations and stakeholders who can provide supportive networks and services, not just during a global crisis but also in general. However, for the benefits of digital space to be fully realized the digital divide between privileged and underprivileged individuals and contexts that already existed before the pandemic and was made worse because of the necessitated shift to online learning would first need to be addressed.

To conclude, approaching research on the development and well‐being of adolescents and emerging adults from a more inclusive and ecological perspective is essential during a pandemic and in general, as this will better inform intervention. The four systematic reviews had an extensive focus on the family, school, and peer contexts as well as social media, communities, and social institutions (micro‐ and exosystem). Partnership between immediate contexts (mesosystem) and the influence of cultural values and social norms (macrosystem) are also important determining factors that can be highlighted in future research. In addition to factors at different levels within young people's ecology, research investigations should consider time‐related elements like educational and developmental transitions as well as the ever‐growing digital space of youth. Within this inclusive scope, it is important to nurture both internal and external developmental assets, ensuring an optimal alignment between these two sets of assets. Capitalizing on the strengths and resources of young people, whether during a pandemic or generally, is better than focusing on their weakness or what they lack. What promotes thriving and contribution, however, may vary with culture, ethnicity, and other characteristics of youth, nuances that need to be considered to better address the needs of this diverse group. Nonetheless, due to education, modernization, and globalization, young people across the globe have many things in common. Thus, common contexts like digital space can be used to strengthen efforts that do not only favor young people from Western, educated, industrialized, rich, and democratic (WEIRD) countries but also youth across the non‐WEIRD majority world.

## FUNDING INFORMATION

No funding was received for this manuscript.

## CONFLICT OF INTEREST STATEMENT

The authors declare no conflicts of interest.

## Data Availability

Data sharing is not applicable to this article as no datasets were generated or analyzed during the current study.
